# A Structured Health Reception Programme in Primary Care for Asylum Seekers: Preliminary Clinical, Preventive, Service-Use and Psychosocial Findings

**DOI:** 10.3390/healthcare14213791

**Published:** 2026-11-06

**Authors:** Sara Louise Gómez-Langley, Ainhoa Ruiz-Azarola, Henry Antonio Andrade-Ruiz

**Affiliations:** 1 Torreblanca Primary Care Centre, Seville Primary Care Health District, Andalusian Health Service, Andalusia, 41016 Seville, Spain; 2 Andalusian School of Public Health, Cuesta del Observatorio 4, Andalusia, 18011 Granada, Spain; ainhoa.ruiz.easp@juntadeandalucia.es; 3 Instituto de Investigación Biosanitaria ibs.GRANADA, Andalusia, 18011 Granada, Spain; 4 Methodological and Statistical Management Unit, Foundation for Health Research Management (FISEVI), Andalusia, 41013 Seville, Spain; hantonio.andrade@juntadeandalucia.es

**Keywords:** international protection, primary healthcare, health disparities, health literacy, vaccination, anemia, infectious disease screening

## Abstract

**Highlights:**

**Public health relevance: How does this work relate to a public health issue?**

• Refugees and asylum seekers may have heterogeneous health needs and may face difficulties finding their way through an unfamiliar health system.
• A structured primary-care reception pathway can organize the initial assessment, support understanding of the health system, and facilitate transition to routine care.

**Public health significance: Why is this work of significance to public health?**

• The PAS (Programa de Acogida Sanitaria, PAS) identified preventive vaccination needs and relevant infectious findings through distinct clinical assessment processes.
• Hematological findings identified additional clinical needs and support a broader reception approach than infectious-disease screening alone.

**Public health implications: What are the key implications or messages for practitioners, policy makers and/or researchers in public health?**

• The PAS combines risk-adapted assessment, prevention, understandable communication, teletranslation when needed, coordinated review, and continuity with routine care.
• Organizational health literacy responsiveness should be distinguished from individual health literacy, as the latter was not directly assessed in this study. The organizational capacity of the pathway to provide understandable, accessible, and culturally responsive care requires specific evaluation in future studies.

**Abstract:**

Background/Objectives: Asylum seekers may have heterogeneous clinical and preventive needs and face difficulties in finding their way through an unfamiliar health system. This study reports a preliminary cross-sectional analysis of a structured Health Reception Programme (Programa de Acogida Sanitaria, PAS) embedded in primary care within an ongoing mixed-methods project. Methods: The study was conducted in Seville, Spain, among participants whose initial PAS consultations took place between January and October 2025, with an interim analytical database cut-off in September 2026. The cohort included 122 participants. Results: Relevant infectious processes, vaccination needs, anemia, iron status, extended PAS use, and migratory grief were assessed. A relevant infectious process was detected in 26/121 participants (21.5%; 95% CI 15.1–29.6), and at least one vaccination need in 74/120 (61.7%; 52.7–69.9). Anemia was recorded in 12/122 (9.8%; 5.7–16.4), while 56/120 (46.7%; 38.0–55.6) had non-normal iron status, including 41 participants classified as having iron deficiency. Of the 12 participants with anemia, 11 were classified as having iron deficiency, whereas 30 of the 41 participants with iron deficiency did not have anemia. Female sex was associated with anemia, whereas increasing age was associated with lower odds of non-normal iron status. Thirty-five participants (28.7%) had four or more consultations. Psychosocial findings were exploratory. Conclusions: Overall, the findings support a prevention-oriented, risk-adapted primary-care reception pathway that identifies clinical needs beyond infectious-disease screening. Organizational health-literacy responsiveness requires direct evaluation.

## 1. Introduction

At the end of 2025, 117.8 million people worldwide were forcibly displaced because of persecution, conflict, violence, human-rights violations, or events seriously disturbing public order; 41.6 million were refugees and 9.0 million were asylum-seekers [1]. Forced displacement and migration, however, are not clinical diagnoses, and people crossing borders do not constitute a homogeneous risk group. Health across the migration trajectory is shaped by circumstances in countries of origin, exposures during travel, legal and social conditions after arrival, access to preventive services, and the responsiveness of receiving health systems [2,3]. Framing migration primarily through infectious risk can therefore obscure both heterogeneity and the wider structural conditions that generate avoidable health inequalities.

European evidence consistently describes barriers to equitable care among migrants, refugees and asylum-seekers, including language and communication difficulties, administrative restrictions, discrimination, unfamiliarity with how the health system works, and unmet preventive and mental-health needs [4]. Recent work in primary care likewise argues that equitable assessment requires culturally responsive, system-level approaches that make health needs visible without reducing them to a narrow biomedical problem [5]. The World Health Organization (WHO) has described generalist, specialized focus, gateway, and limited models through which health systems respond to refugee and migrant health needs [6]. On this basis, the Health Reception Programme (Programa de Acogida Sanitaria, PAS) examined in this study functions as a structured entry pathway embedded in routine primary care, followed by transition to general services.

Infectious-disease screening is one component of such care, but international guidance supports targeted rather than indiscriminate testing. The European Centre for Disease Prevention and Control recommends evidence-based screening and vaccination strategies tailored to epidemiology, country or region of origin, age, and other relevant circumstances [7]. Spanish primary-care recommendations likewise advocate individualized assessment for latent tuberculosis infection (LTBI), viral hepatitis, selected parasitic infections, sexually transmitted infections, mental health, and female genital mutilation according to epidemiological and clinical context [8,9]. This approach is particularly relevant in low-incidence settings, where migration-related tuberculosis burden is often driven by remotely acquired LTBI rather than recent transmission [10].

Vaccination is also a central component of preventive equity. WHO recommends explicit inclusion of refugees and asylum seekers in national immunization policies and catch-up strategies across the life course [11]. European reviews identify barriers related to access, awareness, acceptance, activation, communication, documentation, and service organization [12]. Meta-analytic evidence further indicates that vaccination coverage among migrants is heterogeneous and often suboptimal [13].

Nutritional and hematological needs are another potentially under-recognized component of comprehensive reception care. Findings such as anemia or non-normal iron status can have diverse causes, including inadequate intake or absorption, blood loss, infection or inflammation, chronic disease, or inherited disorders; their clinical meaning depends on age, sex, and individual context rather than on migration status itself [2].

Beyond clinical needs, the way health systems organize access and communication shapes people’s ability to use health services. Health literacy refers to the knowledge and capabilities needed to access, understand, appraise, and use health information and services [14]. However, in migrant populations, it cannot be inferred from educational attainment, language, or country of origin [15]. Organizational health-literacy responsiveness is therefore particularly relevant: services have a responsibility to make information, procedures, and care pathways easy to find, understand, and use for people with diverse needs [16]. Among refugee populations, access to interpreting, continuity of care, culturally responsive communication, and support in using the health system have been identified as components of more accessible and equitable primary care [17,18]. From this perspective, health literacy arises from the interaction between people’s capabilities and the demands of the system rather than representing an individual deficit attributable to origin [19,20].

Psychosocial needs also form part of a comprehensive reception pathway. Post-migration stressors, including prolonged uncertainty, social isolation, discrimination, family circumstances, unemployment, and communication difficulties, are associated with mental-health outcomes among refugees and asylum seekers populations [21]. Language support and culturally safe communication are particularly important in migrant mental healthcare [22]. In Spanish primary care, De la Revilla et al. developed a 17-item questionnaire identifying four dimensions of migratory grief—fear, nostalgia, concern, and loss of identity—providing a framework for exploring psychosocial experiences without assuming a single uniform syndrome [23].

Against this background, a structured health reception programme, PAS, has been operating since 2018 in a primary-care centre in Seville for people living in a reception facility serving asylum seekers. The programme combines an initial joint physician–nurse consultation, culturally responsive history taking, testing selected according to origin and clinical risk, vaccination review, planned follow-up, and referral when indicated. This article reports the preliminary quantitative cut of the ongoing mixed-methods project AP-0556-2024 C5-F1. The primary objective was to characterize clinical and preventive needs identified through the PAS and describe how they varied across sociodemographic and migration-related characteristics. Secondary objectives explored pathway use and dimensions of migratory grief. Because no validated health-literacy instrument was administered, the manuscript examines only organizational features of the PAS consistent with health-literacy-responsive care and does not assign individual health-literacy levels to participants.

## 2. Materials and Methods

### 2.1. Study Design and Setting

This study presents an interim descriptive analysis of observational data prospectively collected within the quantitative component of an ongoing mixed-methods research project (AP-0556-2024 C5-F1). The Health Reception Programme (Programa de Acogida Sanitaria, PAS) is delivered at Torreblanca Primary Care Centre in Seville, Spain, and provides structured primary healthcare assessments for individuals accommodated at a reception facility operated by the Spanish Commission for Refugee Aid (CEAR).

The parent study covers 2025–2026, with a planned sample of approximately 300 participants. The present analysis includes 122 participants whose first recorded PAS consultations occurred between January and October 2025. The analytical database was locked in September 2026, following the extraction, pseudonymization, and review of clinical information available for this analysis.

This report provides an initial description of the health needs identified and healthcare utilization patterns observed among individuals assessed through the PAS. The ongoing parent study will incorporate additional participant records from the 2025–2026 study period, enabling more comprehensive analyses of the programme once the planned sample has been reached.

The PAS follows an individualized care pathway in which the number of consultations is determined by each participant’s clinical needs and previous healthcare assessments. For individuals without prior evaluations, the usual initial assessment pathway comprises three consultations. However, participants who have already undergone clinical examinations or diagnostic investigations at other healthcare facilities may require fewer consultations, whereas those with additional clinical or preventive needs may require further visits. Thus, three consultations represent the usual care pathway rather than a mandatory requirement for programme completion.

The present analysis describes clinical conditions, preventive healthcare needs, and healthcare contacts documented during PAS assessments. As some participants remained under follow-up, the number of recorded consultations should not be interpreted as a measure of programme completion, adherence, or discontinuation of care. The findings do not represent a longitudinal assessment of clinical outcomes or establish the causal effectiveness of the programme [24].

### 2.2. PAS Care Pathway

The PAS is integrated into routine primary care and provides an individualized health assessment based on each participant’s clinical history, previous healthcare evaluations, and identified needs. The programme seeks to complete the initial assessment in as few consultations as necessary. For individuals who have not undergone previous healthcare assessments, the usual pathway consists of three consultations, although this may vary according to individual circumstances.

The first consultation is conducted jointly by a physician and a nurse. It includes a clinical history, physical examination, measurement of vital signs, review of available medical records, and assessment of vaccination status and current living conditions. Particular attention is paid to the participant’s country and geographical area of origin, rural or urban background, migration route, countries crossed, travel conditions, time spent in Spain, previous illnesses and treatments, and preventive healthcare history. Language and communication needs are also assessed, and teletranslation is available when needed. Blood samples are collected, tuberculin skin testing (Mantoux test) is performed when indicated, and additional investigations are requested according to epidemiological and clinical risk.

The second consultation is usually conducted by nursing staff and may include Mantoux test reading, collection of biological samples, and coordination of additional investigations, including imaging when indicated. At the third consultation, the physician reviews laboratory and imaging results, discusses the findings with the participant, initiates or coordinates treatment and preventive measures, and arranges further investigations or referrals when necessary. Clinical findings and decisions are documented, after which participants continue receiving care through routine primary care services.

The number of consultations is adjusted according to each participant’s needs. Those who have already undergone clinical assessments or diagnostic investigations at other healthcare facilities may require fewer visits, while additional consultations may be needed to address further clinical or preventive issues. Therefore, the number of recorded consultations alone cannot be used to determine whether the initial assessment was completed or whether a participant discontinued care, are reported in Supplementary Table S11.

Diagnostic testing is selected according to geographical origin, epidemiological exposures, clinical findings, and individual risk factors rather than being applied uniformly to all participants. This should be considered when interpreting the number of participants assessed for each condition and the corresponding diagnostic findings.

### 2.3. Participants and Preliminary Analytical Cohort A

A total of 250 individuals had been attended by the Spanish Commission for Refugee Aid (CEAR). According to information confirmed by CEAR, all individuals attended through this reception facility were asylum seekers. Individual legal or administrative protection status was not separately recorded as a variable in the analytical dataset. Participants were referred to the PAS by CEAR, which organized and scheduled clinical interviews on a weekly basis. The study clinician assessed individuals according to this referral and appointment schedule; participant selection was therefore not independently determined by the clinician.

At the time of the September 2026 interim database lock, records from 122 participants (48.8%) had been consecutively incorporated into the research database, following the chronological order of their first recorded PAS consultations from January 2025 onwards, and were available for analysis. The remaining 128 records (51.2%) had not yet been entered into the database and were therefore not included in the present analysis. These pending records were not classified as exclusions, refusals, or losses to follow-up. Thus, the interim cohort reflects the chronological incorporation of records from individuals referred to and assessed through the programme, rather than a selection based on clinical characteristics.

The 122 participants included in the interim cohort had their first recorded PAS consultations between January and October 2025. Completion of the usual three-consultation pathway was not required for inclusion, as the number of visits was individualized according to previous healthcare assessments and clinical needs.

The composition of the interim cohort and the records pending database entry are presented in Supplementary Figure S1. Outcome-specific evaluable denominators are reported in Supplementary Table S12.

### 2.4. Variables, Main Outcomes, and Secondary Clinical Findings

Sociodemographic and migration-related variables included age, sex as recorded in the clinical dataset, geographical area of origin, rural or urban background, travel with or without family, educational level, main language, direct or indirect migration route, travel duration, time in Spain, and number of PAS consultations. Education and main language were analyzed as social and communication-related characteristics and were not interpreted as measures of individual health literacy.

Time in Spain was obtained through participant self-report during the first PAS consultation, when individuals were asked how long they had been living in Spain. This variable therefore reflects the reported duration of residence in Spain at the initial PAS assessment, rather than the time elapsed until the analytical database lock. Importantly, access to the PAS is linked to the reception pathway at CEAR Torreblanca rather than to the time elapsed since arrival in Spain. Time registered with CEAR Torreblanca was considered a separate variable reflecting the duration of registration with the reception organization and providing a more relevant temporal context for entry into the PAS.

The duration of the PAS process was calculated as the interval, in days, between the date of the first recorded PAS consultation and the date of administrative closure of the programme, documented by the issuance of the final health report. Administrative closure does not necessarily imply that all planned clinical investigations had been completed, as the programme could be closed when a participant was no longer registered as a user of the primary care centre. Duration was summarized using the median, interquartile range (IQR), and full range. The calculation was performed for participants with both dates available.

Infectious disease screening within the PAS was guided by recommendations from the World Health Organization (WHO), the European Centre for Disease Prevention and Control (ECDC), and the clinical protocols of the referral hospitals. These recommendations consider the epidemiological characteristics of countries of origin, particularly the prevalence and endemicity of specific infections, together with individual clinical and epidemiological risk factors.

Countries of origin were grouped into geographical regions for descriptive and statistical analyses. However, diagnostic investigations were selected according to the epidemiological characteristics of each participant’s country of origin, migration history, previous healthcare assessments, and clinical findings, rather than geographical grouping alone.

Consequently, screening was not uniform across participants. The number of individuals tested varied according to the infection under investigation, and positivity estimates were calculated using the number of participants assessed for each condition. Testing coverage and positive findings for individual infectious conditions are presented in Supplementary Table S6.

Diagnostic investigations were requested within the PAS according to individual clinical and epidemiological indications. Biological samples were sent to Hospital Universitario Virgen del Rocío (Seville, Spain) for processing and analysis by the corresponding hospital laboratory services.

Latent tuberculosis infection (LTBI) was assessed using the tuberculin skin test (Mantoux) and/or an interferon-gamma release assay (IGRA). The Mantoux test was routinely performed in individuals younger than 65 years, while testing in those older than 65 years was considered when they originated from specific endemic areas. Chest radiography was performed when the Mantoux induration ≥ 5 mm. Among migrants who had lived in Spain for fewer than five years, a tuberculin skin test was considered positive when the induration diameter ≥ 10 mm, with a lower threshold of ≥5 mm applied to children younger than five years and immunocompromised individuals. In participants with a Mantoux induration ≥ 10 mm who had a history of Bacillus Calmette–Guérin (BCG) vaccination, or whose vaccination history was uncertain, IGRA testing was considered. Classification as LTBI required the absence of clinical or radiological evidence of active tuberculosis.

Hepatitis B infection was assessed using serological markers, including hepatitis B surface antigen (HBsAg) and hepatitis B core antibodies (anti-HBc). Additional serological markers and HBV DNA testing were performed when clinically indicated. Acute hepatitis B infection was defined by the concomitant presence of HBsAg and positive IgM anti-HBc antibodies. Chronic HBV infection was distinguished from active hepatitis based on the persistence of HBsAg, the presence of IgG anti-HBc antibodies, and additional virological and clinical findings, including HBV DNA levels, liver enzyme levels, and evidence of liver damage.

Schistosoma testing was requested according to the participant’s country of origin and included serological and/or parasitological methods. Detection of characteristic eggs in stool or urine was considered evidence of active infection, whereas positive serology was interpreted as evidence of exposure or possible infection requiring clinical assessment by the infectious diseases service of the referral hospital.

Stool parasitology was requested originating from high-risk areas who had been living in Spain for less than one year, with treatment prescribed according to the findings. For those who had been living in Spain for more than one year, the clinical approach was similar to that applied to the native population. Among migrants from countries not classified as high risk, parasitological testing and treatment were considered when additional risk factors were present, including overcrowding, contact with infected individuals, or cohabitation with people originating from high-risk countries. Stool parasitology was assessed through microscopic examination, concentration methods, and/or molecular testing, as applicable. Positive findings were based on the identification of pathogenic intestinal parasites or detection of their genetic material. Non-pathogenic protozoa alone were not considered clinically relevant parasitic infections.

Mycoplasma testing was included in the diagnostic work-up when a sexually transmitted infection (STI) was suspected and was not performed systematically. Testing was based on molecular detection using nucleic acid amplification methods. A positive result was defined by detection of the corresponding organism in an appropriate clinical specimen, interpreted in conjunction with the patient’s clinical presentation.

The first main outcome, relevant infectious process detected, was defined as the presence of at least one of the following findings within the PAS pathway: latent tuberculosis infection (LTBI), HIV infection, active hepatitis B, positive Schistosoma testing, positive stool parasitology, or a sexually transmitted infection (STI). STI testing included testing for *Neisseria gonorrhoeae*, *Chlamydia trachomatis*, *Trichomonas vaginalis*, and *Mycoplasma urealyticum*. These tests were requested selectively according to clinical suspicion of an STI and were not part of routine screening for all participants. In the present cohort, the only positive STI result recorded was for *M. urealyticum*. The term “relevant infectious process detected” was used because LTBI does not represent active tuberculosis. Individual components were not mutually exclusive. A participant was classified as negative when interpretable information was available and none of the assessed components was positive. Participants without interpretable information were considered non-classifiable. Given the risk-adapted screening strategy, this outcome describes infections documented during the PAS assessment rather than population prevalence.

The second main outcome, any vaccination need identified, was assessed according to the vaccination schedule established by the Regional Ministry of Health, Presidency and Emergencies of the Regional Government of Andalusia. In accordance with this schedule, the analytical outcome was restricted to routinely recommended vaccinations against hepatitis B, measles, mumps and rubella (MMR), and varicella. It was classified as positive when at least one of these three vaccines was indicated. Absence of vaccination need was assigned only when all three indications were evaluable and none was indicated. Tests or assessments that were not requested, not performed, or had missing results were not treated as evidence of no vaccination need. Tetanus–diphtheria–polio vaccination was assessed separately within the PAS. Following examination of the observed data, tetanus–diphtheria–polio vaccination was excluded from the composite outcome because an indication was recorded in 92.6% of participants. Including this highly prevalent indication would have caused the composite to be largely determined by tetanus–diphtheria–polio vaccination needs, limiting its ability to describe variation in hepatitis B, MMR, and varicella vaccination indications. The analytical composite was therefore restricted to these three vaccines. This restriction was made during the analysis and was not prespecified. Other risk-group vaccine indications were also excluded from the composite. The corresponding evaluation of vaccination needs is presented in Supplementary Table S9.

The third main outcome, extended PAS utilization, was defined as four or more consultations versus three or fewer. For participants without previous healthcare assessments, the usual PAS pathway consists of three consultations. However, the number of visits is individualized according to previous evaluations and clinical or preventive needs, as described in Section 2.2. Therefore, four or more consultations were considered a descriptive indicator of healthcare utilization rather than inappropriate or excessive use, while fewer consultations did not necessarily indicate incomplete assessment or discontinuation of care. The total number of consultations was also analyzed as a count outcome in a sensitivity analysis. The distribution of consultations and the corresponding migration-related time variables are presented in Supplementary Table S10.

Two secondary clinical findings were analyzed: anemia and iron status. Anemia was extracted as a binary clinical variable (yes/no) from the participants’ medical records, based on the interpretation of hemoglobin results using the age- and sex-specific reference ranges applied by the Hospital Universitario Virgen del Rocío laboratory. The clinical classification was retained as recorded and was not independently recalculated using individual hemoglobin values or standardized WHO thresholds in the present analysis.

Iron-status categories were obtained from the original clinical classifications recorded in the study database. Iron deficiency was classified on the basis of serum ferritin concentrations, using the reference intervals provided by the clinical laboratory. These categories were retained as documented and were not independently reassessed using predefined diagnostic criteria. The non-normal iron-status category comprised participants classified as having minor iron-related alterations or iron deficiency. Laboratory reference intervals for hemoglobin and iron-related parameters are provided in Supplementary Table S8.

This grouping was used to describe associations with any recorded deviation from normal iron status, without assuming that minimal alteration necessarily represented established iron deficiency. Anemia and iron status were also cross-tabulated, but the recorded classification of iron deficiency was not considered sufficient, by itself, to establish iron-deficiency anemia. The distribution of the original categories and their relationship with anemia are presented in Supplementary Table S7.

### 2.5. Migratory Grief: Instrument and Scoring

Migratory grief was assessed in adults using the 17-item questionnaire developed by De la Revilla et al. [23]. The original exploratory factor analysis identified four dimensions explaining 52.1% of total variance. Following the factor structure, fear comprised items 12–17 (theoretical range 0–12), nostalgia items 1, 4, 5, 6, and 7 (0–10), concern items 2, 8, 9, and 11 (0–8), and loss of identity items 3 and 10 (0–4). Dimension scores were calculated only when all items for that dimension were available. Because the original publication did not provide a single numeric recoding scheme for the mixture of binary and three-category responses, an a priori operational recoding placed all items on a 0–2 scale: never = 0, sometimes = 1, always = 2 for ordinal items; no = 0 and yes = 2 for direct binary items; and the identification-with-compatriots item (item 10) was reverse-coded as yes = 0 and no = 2. Item 7 was included in the nostalgia dimension because it loaded on that factor in the published factor-loading table. Higher scores represent greater expression of the corresponding dimension. Loss of identity was interpreted cautiously because the original validation reported very low internal consistency for that factor. The questionnaire was originally developed and validated in a Spanish primary care immigrant population [23]. The present study used the established instrument to describe migratory grief among adult PAS participants and did not undertake a new psychometric validation.

### 2.6. Statistical Analysis

Continuous variables were summarized using median and interquartile range (IQR) and, when informative, mean and standard deviation (SD); categorical variables were summarized as counts and percentages using explicit outcome-specific denominators. Binomial 95% confidence intervals (CIs) for the main proportions were calculated using the Wilson method. Missing or non-evaluable information was not recorded as a negative result.

The infectious outcome model was prespecified to include age group (<18 versus ≥18 years), sex, and geographical area. Because of the limited event count, sparse strata, and the possibility of separation, adjusted associations were estimated using Firth penalized logistic regression with profile penalized likelihood 95% CIs [25,26]. Sub-Saharan Africa, age < 18 years, and male sex were the reference categories. A sensitivity analysis replaced the dichotomous age term with age per 10-year increase. A context model replacing geographic area with selected migration-related characteristics was used as a secondary analysis. Covariates were not selected according to univariable *p*-values.

An additional post hoc sensitivity analysis restricted the infectious composite to latent tuberculosis infection, active hepatitis B, and HIV infection. The original and restricted composites were compared using Firth penalized logistic regression adjusted for age group, sex, and geographical area. Both models were fitted to the same participants for whom all three components of the restricted composite were evaluable, allowing direct comparison of the geographical associations.

Vaccination needs were treated primarily as descriptive and exploratory. The composite and vaccine-specific proportions were reported, and bivariate comparisons were performed across age, sex, geographic area, rurality, family accompaniment, education, main language, and time in Spain. Fisher exact tests were used for categorical comparisons when appropriate, and time in Spain was retained as a continuous variable and compared using the Wilcoxon rank-sum test.

Anemia and iron status were summarized using explicit evaluable denominators and Wilson 95% CIs. Exploratory bivariate associations with sex, age group, and geographical area were assessed using Fisher exact tests when appropriate; age was also examined continuously with Wilcoxon rank-sum tests. Adjusted associations were estimated separately for anemia and non-normal iron status using Firth penalized logistic regression with age per 10-year increase, sex, and geographical area entered a priori. These hematological analyses were considered secondary and exploratory, with particular caution for sparse geographical strata.

The principal model for extended PAS utilization included age group, sex, geographical area, family accompaniment, and time in Spain in six-month units and used Firth logistic regression because separation had been observed in conventional logistic models. As a sensitivity analysis, the number of consultations was analyzed using Poisson regression with heteroskedasticity-consistent (HC3) standard errors. Infectious detection and vaccination need were not included in the primary utilization model because they may arise during the care pathway and could lie downstream of the service process.

Migratory-grief dimensions were analyzed as numeric outcomes using linear regression with HC3 robust standard errors. To avoid overfitting and unstable coefficient reversal among strongly related migration characteristics, three exposure-specific model sets were prespecified: family accompaniment, educational level (secondary/university versus none/primary), and geographical area (Latin America versus sub-Saharan Africa). Each model adjusted for sex, adult age per 10 years, and time in Spain per six months. Sensitivity analyses for accompaniment and education additionally adjusted for geographic area in the sub-Saharan Africa/Latin America subset. These psychosocial analyses were considered exploratory; no multiplicity adjustment was applied, and interpretation emphasized effect size and 95% CI rather than isolated *p*-values.

Analyses were performed in R version 4.5.2 (R Foundation for Statistical Computing, Vienna, Austria) using packages including haven, dplyr, gtsummary, logistf, sandwich, and lmtest [27]. All tests were two-sided.

### 2.7. Ethics and Data Governance

The study was conducted in accordance with the Declaration of Helsinki and received a favourable opinion from the Research Ethics Committee of Virgen del Rocío University Hospital (application/communication code SICEIA-2024-002745; study code DSAP_S3476_1; approval date: 15 November 2024). Written informed consent was obtained before participation. Information was provided in an understandable manner, with teletranslation or language support available when needed. Access to study data was restricted to authorized researchers in accordance with Regulation (EU) 2016/679 and Spanish Organic Law 3/2018.

## 3. Results

### 3.1. Participant Characteristics

Of the 250 individuals attended by CEAR, 122 (48.8%) had been consecutively incorporated into the study database at the September 2026 interim analytical cutoff. The remaining 128 records (51.2%) were pending database entry. The participant flow is presented in Supplementary Figure S1.

The preliminary analytical cohort comprised 122 participants. Median age was 26 years (IQR 19–33; range 1–77; mean 27.1 ± 14.2), and 98 (80.3%) were adults. Eighty-seven participants (71.3%) were male and 35 (28.7%) female. Sub-Saharan Africa was the most frequently represented geographical area (52/122, 42.6%), followed by Latin America (42/122, 34.4%), Eastern Europe (15/122, 12.3%), Asia (10/122, 8.2%), and the Maghreb (3/122, 2.5%).

The most frequently recorded countries of origin were Venezuela (31/122, 25.4%), Mali (25/122, 20.5%), Senegal (18/122, 14.8%), Ukraine (15/122, 12.3%), and Palestine (9/122, 7.4%). The complete distribution of recorded countries and territories of origin is presented in Supplementary Table S13.

Thirty-five participants (28.7%) came from rural settings, 72 (59.0%) had travelled without family, and 74 (60.7%) had followed an indirect migration route. Spanish was the main language for 42 (34.4%) and another language for 80 (65.6%). Among the 103 participants with education recorded, 52 (50.5%) had no schooling or primary education and 51 (49.5%) had secondary or university education. At the first PAS consultation, the self-reported duration of residence in Spain had a median of 11 months (IQR 7–15). Travel duration was markedly right-skewed, with a median of 1.0 month (IQR 0.1–24.0) despite a mean of 16.5 ± 27.8 months.

Three consultations were the most frequently recorded number of PAS contacts (66/122, 54.1%). Five participants (4.1%) had one consultation, 16 (13.1%) had two, and 35 (28.7%) had four or more. The number of consultations varied according to previous healthcare assessments and individual clinical needs and was not used to determine whether participants had completed or discontinued care. The distribution of consultations is presented in Table 1.

The duration of the PAS process could be calculated for 120 of the 122 participants, as two records had incomplete date information. The median duration was 28 days (IQR: 14–42; range: 0–294), with a mean of 39.17 days. Five participants had an interval of 0 days between their first recorded PAS consultation and administrative closure of the programme.

### 3.2. Main Quantitative Outcomes

Among 121 participants classifiable for the infectious composite, 26 had at least one relevant infectious process detected (21.5%; 95% CI 15.1–29.6). Among 120 participants classifiable for vaccination need, 74 had an indication for at least one of hepatitis B, MMR, or varicella vaccination (61.7%; 95% CI 52.7–69.9). Thirty-five of 122 participants had four or more PAS consultations (28.7%; 95% CI 21.4–37.3). As secondary clinical findings, anemia was recorded in 12/122 participants (9.8%; 95% CI 5.7–16.4), and 56/120 participants with interpretable iron assessment had a non-normal iron status (46.7%; 95% CI 38.0–55.6). The frequencies of the main and secondary clinical outcomes are presented descriptively in Table 2, recognizing that their ascertainment procedures and evaluable denominators differed. Outcome-specific data availability and the corresponding numbers of evaluable and non-evaluable participants are presented in Supplementary Table S12.

### 3.3. Detection of Relevant Infectious Processes

LTBI was the predominant component of the infectious composite (n = 19), followed by active hepatitis B (n = 4) and positive *Schistosoma* testing (n = 3); one HIV infection, one positive stool-parasitology result, and one *M. urealiticum* infection were also identified. Components were not mutually exclusive. Because testing was selected according to epidemiological and clinical risk, the 21.5% composite estimate describes documented detection within the PAS rather than the prevalence of infection in the underlying asylum seeker population. Hepatitis C and syphilis were also considered among the infectious conditions recorded within the parent research project. No positive cases of these infections were identified in the present interim analytical cohort of 122 patients. Screening for Strongyloides, as stipulated by current European recommendations for migrants arriving from endemic areas, was not performed, as Strongyloides serology cannot be requested through primary care at our reference laboratory. This represents a limitation in the comprehensive screening for infectious diseases among asylum seekers.

In unadjusted Firth models, several characteristics appeared to be associated with infectious detection: adults had higher odds than minors (OR 5.51; 95% CI 1.31–51.15), rural background had higher odds than urban background (OR 4.27; 1.75–10.70), and use of a main language other than Spanish was associated with higher odds (OR 3.35; 1.21–11.28). Conversely, travelling with family (OR 0.15; 0.04–0.45) and secondary/university education (OR 0.28; 0.10–0.71) were associated with lower crude odds. These characteristics were strongly interrelated with geographic area and therefore were not interpreted as independent causal determinants (Supplementary Table S1).

In the prespecified adjusted model including age group, sex, and geographic area, the crude age association was substantially attenuated (aOR for adults 1.79; 95% CI 0.30–19.09; *p* = 0.546), and sex was not independently associated with the outcome (aOR for female sex 2.17; 0.58–9.33; *p* = 0.249). Compared with participants from sub-Saharan Africa, those from Latin America had lower odds of a relevant infectious process being detected (aOR 0.15; 0.03–0.55; *p* = 0.003). The estimate for Asia was also lower (aOR 0.08; 0.00–0.96; *p* = 0.045), but the small subgroup size and sparse events warrant particular caution. Estimates for the Maghreb and Eastern Europe were imprecise and inconclusive (Table 3). The number of participants tested and the corresponding positive findings for each infectious condition, stratified by geographical region, are presented in Supplementary Table S6.

In a post hoc sensitivity analysis restricting the infectious composite to latent tuberculosis infection, active hepatitis B, and HIV, 22 of 121 classifiable participants (18.2%) had at least one positive finding, compared with 26 of 121 (21.5%) for the original composite. Among the 111 participants with all three components evaluable, 21 (18.9%) had a positive restricted composite. The adjusted geographical association persisted when both outcomes were evaluated in this common analytical sample. Compared with sub-Saharan Africa, participants from Latin America had lower odds of infectious detection using both the original composite (aOR 0.15; 95% CI 0.03–0.54; *p* = 0.003) and the restricted composite (aOR 0.08; 95% CI 0.01–0.36; *p* < 0.001). Testing coverage, geographical distributions, and adjusted estimates are presented in Supplementary Table S16.

When age was parameterized continuously, there was again no clear independent age association (aOR per 10 years 1.30; 0.90–1.93; *p* = 0.162), whereas the Latin America versus sub-Saharan Africa contrast remained materially unchanged (aOR 0.11; 0.02–0.40; *p* < 0.001). In an alternative context model excluding geographic area, rural background retained a positive but imprecise association (aOR 2.14; 0.80–5.94), and travel with family showed an inverse but uncertain association (aOR 0.23; 0.03–1.06).

### 3.4. Vaccination Needs

Vaccination need was the most frequent actionable outcome. At least one indication for hepatitis B, MMR, or varicella vaccination was identified in 74/120 participants (61.7%; 95% CI 52.7–69.9). Hepatitis B vaccination was indicated in 54/121 (44.6%; 36.1–53.5), MMR in 37/120 (30.8%; 23.3–39.6), and varicella in 29/120 (24.2%; 17.4–32.6). The evaluation of the composite vaccination outcome is presented in Supplementary Table S9.

Exploratory bivariate analyses did not show clear heterogeneity in the composite vaccination need by sex, geographic area, rurality, family accompaniment, education, or main language. Vaccination need was descriptively more frequent among participants younger than 18 years (19/24, 79.2%) than among adults (55/96, 57.3%), although the comparison did not reach conventional statistical significance (*p* = 0.061). Median time in Spain was 12 months among those without a vaccination need and 10 months among those with a need (*p* = 0.14). These comparisons were considered descriptive and were not escalated into a multivariable vaccine model in the preliminary analysis. Exploratory bivariate associations are presented in Supplementary Table S2, while the evaluability and classification of the composite vaccination outcome are detailed in Supplementary Table S9.

### 3.5. Hematological Findings

Anemia was recorded in 12/122 participants (9.8%; 95% CI 5.7–16.4). It was more frequent among women than men [7/35 (20.0%; 95% CI 10.0–35.9) vs. 5/87 (5.7%; 2.5–12.8); *p* = 0.037]. No clear difference was observed by age group [4.2% in participants <18 years vs. 11.2% in adults; *p* = 0.457], and median age was 26 years in both participants with and without anemia (*p* = 0.860). Geographical heterogeneity was not statistically convincing in the bivariate analysis (*p* = 0.329).

In the Firth model adjusted for age, sex, and geographical area, female sex remained associated with anemia (aOR 4.75; 95% CI 1.23–21.26; *p* = 0.023). Age per 10-year increase was not independently associated (aOR 1.06; 0.67–1.58; *p* = 0.802), and none of the geographical contrasts was conclusive. Because only 12 anemia events were observed, the sex estimate was imprecise and is interpreted as a secondary signal rather than a precise effect-size estimate.

Among 120 participants with interpretable iron assessment, 56 (46.7%; 95% CI 38.0–55.6) had a non-normal status; this group comprised 41 records classified as iron deficiency and 15 as minimal alteration. Non-normal iron status was similar in women and men (48.6% vs. 45.9%; *p* = 0.842) but was substantially more frequent in participants younger than 18 years than in adults (75.0% vs. 39.6%; *p* = 0.003). Participants with a normal iron status were also older on a continuous scale [median 29.0 years (IQR 23.0–36.2) vs. 23.5 years (11.8–28.0); *p* < 0.001] (Supplementary Table S5).

In the adjusted Firth model, each 10-year increase in age was associated with lower odds of a non-normal iron status (aOR 0.46; 95% CI 0.28–0.67; *p* < 0.001), whereas sex was not independently associated (aOR for female sex 1.41; 0.49–4.18; *p* = 0.527). Bivariate heterogeneity by geographical area was observed (*p* = 0.002), but several strata were small All 10 evaluable Asian participants had a non-normal iron result, resulting in complete separation for this geographical category. Although Firth penalized logistic regression yielded a finite adjusted estimate (aOR 28.34; 95% CI 2.67–3961.68), the absence of outcome variation and the small subgroup size precluded reliable interpretation of this contrast. Accordingly, no substantive inference was drawn from the corresponding *p*-value. Latin America was similar to sub-Saharan Africa (aOR 1.06; 0.38–2.87; *p* = 0.914), while Eastern Europe showed a lower, borderline estimate (aOR 0.24; 0.05–1.03; *p* = 0.055) (Table 4). The distribution of the original iron-status categories and their cross-classification with recorded anemia are presented in Supplementary Table S7. In a sensitivity analysis using categorical age, participants younger than 18 years had higher adjusted odds of non-normal iron status than adults (aOR 7.62; 95% CI 2.10–35.01; *p* = 0.001), consistent with the direction of the main model (Supplementary Table S14).

### 3.6. PAS Utilization

Three consultations were the modal pathway (54.1%); 35/122 participants (28.7%; 95% CI 21.4–37.3) had four or more consultations. In the adjusted Firth model, age, sex, and family accompaniment were not clearly associated with extended utilization. Latin American origin showed a lower point estimate relative to sub-Saharan Africa (aOR 0.29; 0.06–1.11; *p* = 0.072), and time in Spain showed a weak inverse trend per six-month increment (aOR 0.66; 0.38–1.06; *p* = 0.096). The Asia estimate was markedly low (aOR 0.02; 0.00–0.37; *p* = 0.004) but arose from a small subgroup and was not treated as a primary finding.

Importantly, the count sensitivity analysis did not reproduce a robust sociodemographic pattern. Rate ratios for the actual number of consultations were close to the null for age, sex, geographic area, family accompaniment, and time in Spain. The binary ≥ 4-consultation outcome should therefore be interpreted as a pragmatic marker of pathway extension rather than a stable phenotype of healthcare use (Supplementary Table S3).

In an additional sensitivity analysis, time in Spain was replaced by time registered at CEAR in the Firth logistic regression model for four or more PAS consultations. Both specifications were fitted to the same 121 participants. Time registered at CEAR was not clearly associated with extended utilization (aOR per month 1.12; 95% CI 0.70–1.74; *p* = 0.623). Adjusted estimates for the other covariates were generally similar across models, although some geographical contrasts remained imprecise (Supplementary Table S18).

The distribution of PAS consultations and the corresponding recorded duration at CEAR Torreblanca reception facility are presented in Supplementary Table S10.

### 3.7. Migratory Grief

Among the 98 adult participants, 69 (70.4%) provided at least one response to the migratory-grief questionnaire, whereas 29 (29.6%) had no questionnaire responses available. The number of participants with complete data varied across questionnaire dimensions, as shown in Table 5. Questionnaire data availability is summarized in Supplementary Table S12. Respondents were descriptively younger, had spent longer in Spain, and included a higher proportion of participants from sub-Saharan Africa than non-respondents; their baseline characteristics are compared in Supplementary Table S15. Fear and nostalgia showed the highest raw scores relative to their respective scales: fear had a median of 7.0 (IQR 3.0–9.0) on a 0–12 scale and nostalgia 7.0 (6.0–9.0) on a 0–10 scale. Concern had a median of 5.0 (4.0–6.0) on a 0–8 scale, whereas loss of identity was concentrated near the lower end of its 0–4 scale (median 0.0; IQR 0.0–2.0). The four domains were not normalized to a common percentage because their theoretical ranges and psychometric properties differ. Exploratory internal consistency estimates were α = 0.614 for fear, 0.672 for nostalgia, 0.472 for concern, and −0.006 for loss of identity. Domain-specific sample sizes and 95% confidence intervals are presented in Supplementary Table S17. The loss-of-identity domain showed very low internal consistency, consistent with the limited reliability reported for this domain in the original study (Cronbach’s α = 0.16) [23].

Family accompaniment was not independently associated with any grief dimension after adjustment for sex, age, and time in Spain. For fear, travelling with family was associated with an adjusted mean difference of +1.29 points (95% CI −1.70 to 4.29; *p* = 0.392); CIs for the remaining dimensions likewise included the null.

Educational level showed the clearest exploratory signal. Participants with secondary or university education had an adjusted mean fear score 1.82 points higher than those with no schooling or primary education (95% CI −0.10 to 3.74; *p* = 0.063) and a nostalgia score 1.17 points higher (−0.15 to 2.49; *p* = 0.081). n the sensitivity analysis restricted to sub-Saharan African and Latin American participants and additionally adjusted for geographic area, the fear difference increased to +2.68 points (0.70 to 4.66; *p* = 0.009) (Supplementary Table S4). Given the reduced sample size, multiple exploratory comparisons, and lack of a prespecified mechanistic hypothesis, this result was treated as hypothesis-generating rather than confirmation.

After adjustment for sex, age, and time in Spain, no clear difference between Latin American and sub-Saharan African participants was observed for fear, nostalgia, or loss of identity. Concern was lower by 1.14 points in the Latin American group, but the CI included the null (95% CI −2.36 to 0.08; *p* = 0.066) (Table 5).

## 4. Discussion

This preliminary analysis of a structured health reception programme for refugees or asylum seekers embedded in primary care yielded a pattern that is relevant to both health-disparities and organizational health literacy responsiveness. First, a relevant infectious process was detected in approximately one in five evaluable participants, while nearly four in five had none of the composite infectious conditions detected. Second, vaccination indications were frequently documented through preventive assessment, whereas infectious findings depended on risk-adapted testing. Third, secondary hematological assessment revealed anemia in approximately one in ten participants and a non-normal iron status in nearly half of those evaluable, with distinct sex- and age-related patterns. Fourth, three visits constituted the modal PAS pathway, and no robust sociodemographic profile of extended utilization was reproduced across binary and count analyses. Fifth, psychosocial assessment showed prominent fear and nostalgia but limited evidence of stable independent associations with family accompaniment or geographic area. Taken together, these findings suggest that the programme’s public health value is not adequately described as infectious-disease case finding alone; it lies in organizing proportionate screening, identifying preventable and hematological needs, facilitating communication, and linking patients into continuing primary care.

This interpretation is consistent with the broader migration-health literature. Migrant health is shaped by interacting pre-migration, transit, and post-arrival determinants, while barriers to care frequently arise from health-system organization, communication, and legal or administrative conditions rather than from a single individual characteristic [2,3,4]. Contemporary refugee-serving primary care literature similarly emphasizes holistic assessment, culturally safe measurement, and the need to make health needs visible without allowing measurement systems themselves to reproduce inequity [5].

### 4.1. Risk-Adapted Infectious Screening: Clinical Yield Without Indiscriminate Testing

The infectious results support a risk-adapted rather than universal narrative. LTBI accounted for most of the composite detections, a pattern that is clinically plausible in a low-incidence host setting and consistent with evidence that much tuberculosis burden among migrants in high-income countries reflects reactivation of infection acquired before migration [10]. ECDC and Spanish primary-care guidance explicitly recommend tailoring screening to origin, epidemiology, age, and clinical context [7,8,9]. The PAS operationalizes this principle through a structured history of country and region of origin, route, time since arrival, and other exposures before tests are selected.

The geographic findings require careful interpretation. Relative to sub-Saharan Africa, Latin American origin was associated with markedly lower odds of infectious detection in both the primary and continuous-age sensitivity models. This contrast is not evidence that geography is an intrinsic causal property of individuals. Geographic area here is a contextual marker that simultaneously reflects source-population epidemiology, migration histories, and the programme’s own rules for risk-adapted testing. The strong dependence observed between geography and rurality, family accompaniment, education, and main language further demonstrates that these variables are structurally intertwined. In particular, language and geography were nearly perfectly aligned in this cohort, making an ‘independent language effect’ statistically and conceptually untenable. The geographical association persisted in the sensitivity analysis restricted to LTBI, active hepatitis B, and HIV, with the contrast between Latin America and sub-Saharan Africa remaining evident after excluding infections subject to more selective testing. This finding supports the robustness of the observed geographical difference, although residual differences in testing indications and underlying epidemiological characteristics cannot be excluded. The results should therefore be interpreted as differences in infectious conditions detected within the programme rather than population prevalence estimates or causal effects of geographical origin.

Equally informative is the negative result: 78.5% of evaluable participants did not have any component of the infectious composite detected. In a targeted programme, negative screening is not a failure of yield; it is part of proportionate risk management when testing is linked to prevention, treatment, and continuity. This distinction is important migrants’ health, where indiscriminate screening can inadvertently reinforce stigma. The finding therefore supports an approach in which screening is clinically actionable and epidemiologically informed rather than premised on the assumption that migration itself denotes infectious risk.

### 4.2. Vaccination Gaps as a Preventive-Equity Signal

The most common actionable finding was vaccination need. More than 60% of classifiable participants required at least one of hepatitis B, MMR, or varicella vaccination, with hepatitis B need alone affecting 44.6%. This result aligns with WHO’s call to integrate migrants fully into routine and catch-up immunization strategies [11]. European evidence shows that under vaccination is produced by multiple interacting mechanisms—access, awareness, affordability, acceptance, and activation—and is not reducible to individual hesitancy [12]. A meta-analysis of migrant vaccination coverage likewise documents substantial heterogeneity and gaps across settings [13].

Vaccination needs and infectious findings were assessed through different clinical processes and should not be directly compared. Vaccination indications were evaluated as part of preventive care, whereas infectious findings depended on risk-adapted testing. Both outcomes describe different aspects of the healthcare needs identified within the PAS. These findings support incorporating vaccination assessment and catch-up planning into routine primary care alongside clinically indicated infectious disease screening.

The descriptive signal of greater vaccination need among minors should not be over interpreted because the confidence around the between-group comparison remained substantial and the *p*-value was 0.061. More importantly, the lack of strong differences across sex, geographic area, rurality, accompaniment, education, or language suggests that vaccination review should remain broadly integrated into the PAS rather than restricted to a small sociodemographic subgroup.

### 4.3. Organizational Health Literacy Responsiveness and Communication

Individual health literacy was not directly assessed in this study, and language or educational attainment should not be interpreted as proxies for the ability to access, understand, appraise, or apply health information [14,15]. However, organizational health-literacy frameworks recognize that healthcare services can reduce the complexity of accessing and navigating care [16]. Research involving refugees and immigrant populations highlights the importance of understandable communication, interpreter access, culturally responsive practice, and continuity of care [17,18,19,20].

The PAS incorporates several features consistent with this organizational approach, including joint physician–nurse assessment, identification of communication needs, access to teletranslation, coordinated diagnostic and preventive activities, and transition to routine primary care. These features reflect an intention to facilitate access and communication, but their effectiveness in improving organizational health literacy responsiveness literacy or patient experience cannot be established from the present findings. The ongoing qualitative phase, involving patients, CEAR staff, and healthcare professionals, will explore how participants experience the accessibility, comprehensibility, and acceptability of the pathway.

### 4.4. PAS Utilization: Pathway Extension Rather than ‘Overuse

Three visits were the intended and observed modal PAS pathway. This matters because four or more contacts should not be framed as healthcare ‘overuse’. Additional visits may represent clinically appropriate work generated by abnormal findings, unresolved preventive needs, communication requirements, or coordination with other services. The primary adjusted model suggested lower odds of extended utilization in some geographic strata, but these estimates were sensitive to sparse subgroup data and were not reproduced as clearly when the actual number of consultations was analyzed as a count. The median self-reported duration of residence in Spain at the first PAS consultation was 11 months. This indicates that the initial PAS assessment did not necessarily coincide with recent arrival in Spain. The term “initial assessment” refers to the participant’s entry into the structured PAS pathway rather than their first healthcare contact in the country. This distinction should be considered when interpreting the programme’s role in identifying and addressing healthcare needs.

The absence of a robust social profile of consultation count is compatible with a standardized pathway that absorbs heterogeneity through structured workflow. From an equity perspective, that may be desirable: differences in language, origin, or family accompaniment need not translate directly into higher visit counts if the service can flex within a coordinated model. Future work should move beyond visit number to examine time to pathway completion, completed referrals, treatment initiation, vaccine delivery, continuity after PAS, and patient-reported burden.

### 4.5. Migratory Grief and Psychosocial Complexity

The grief questionnaire adds a psychosocial dimension that is often missing from biomedical migrant-health evaluations. Fear and nostalgia were the most prominent raw domains in this cohort, whereas loss of identity was concentrated near zero. These dimensions should not be compared as if they were on the same scale, and the loss-of-identity factor warrants particular caution because the original validation reported weak internal consistency [23].

Post-migration mental health is shaped by multiple interacting factors, including duration and uncertainty of the asylum process, social integration, loneliness, discrimination, employment, and communication problems [21]. Communication barriers in migrant mental-health care operate at systemic, interpersonal, and individual levels, and access to appropriate language support is a recurrent need [22]. Against this background, the absence of an independent association between travel with family and grief scores in our small adult sample should not be taken as evidence that family relationships are unimportant. The variable used here captures accompaniment during migration, which is not equivalent to forced family separation, family functioning, current social support, or fear of separation. Broader evidence links policy-related family separation with psychological distress [28].

The exploratory education signal for fear deserves further study rather than a causal interpretation. In the primary model the estimate was positive but imprecise, and it became larger in a geographically restricted sensitivity analysis. Education may correlate with expectations, occupational loss, legal uncertainty, communication experiences, or unmeasured pre-migration circumstances. It is therefore particularly well suited to qualitative follow-up. It should not be interpreted as evidence that more highly educated participants have lower or higher health literacy.

### 4.6. Hematological and Iron-Status Findings as Additional Clinical Needs

The hematological findings broaden the interpretation of the PAS beyond infectious screening and vaccination. Anemia was present in approximately one in ten participants, while almost half of those with evaluable iron-status data had a non-normal result. These two findings should not be collapsed into a single nutritional diagnosis. WHO emphasizes that anemia has multiple causes—including iron and other micronutrient deficiencies, infection and inflammation, blood loss, chronic disease, and inherited red-cell disorders—and that iron deficiency, although a common nutritional cause, is not synonymous with anemia [29]. The PAS therefore identifies a clinical signal that requires etiological assessment rather than assuming that all anemia is iron-related.

The observed sex pattern for anemia is directionally consistent with refugee-health literature. In a cohort of 787 refugees in Germany, Jablonka et al. reported anemia in 22.5% overall and a higher frequency in females than males (27.1% vs. 20.4%) [30]. In a Canadian cohort of 1032 recently resettled adult refugees, Davidson et al. found substantially higher prevalences of iron deficiency and anemia among women than men (25% vs. 0.8% and 21% vs. 1.8%, respectively), and most anemia among women was attributable to iron deficiency when laboratory and clinically assigned diagnoses were considered [31]. Our lower overall anemia frequency and much wider adjusted CI likely reflect differences in age structure, sex distribution, origin, definitions, and setting. Nevertheless, the persistence of the female association after adjustment suggests that hematological assessment should remain part of comprehensive reception care.

Age showed a different pattern for iron status than for anemia. Three quarters of participants younger than 18 years had a non-normal iron result, and older age remained independently associated with lower odds after adjustment. This direction is compatible with the recognized nutritional vulnerability of displaced children, although direct comparison is limited by differences in outcome definitions. A 2024 systematic review and meta-analysis estimated pooled anemia prevalence at 36.5% among displaced and refugee children, with substantial between-study heterogeneity and the greatest burden in younger children [32].

In the present study, iron deficiency was classified on the basis of serum ferritin concentrations, using the reference intervals provided by the clinical laboratory. However, the broader non-normal iron-status category combined clinically recorded iron deficiency and minor iron-related alterations. Accordingly, the overall 46.7% estimate should not be interpreted as the prevalence of iron deficiency. The cross-classification of anemia and iron status showed that 11 of the 12 participants with anemia (91.7%) were classified as having iron deficiency, whereas one had normal iron status. Conversely, 30 of the 41 participants classified as having iron deficiency (73.2%) did not present with anemia. These findings indicate that recorded iron deficiency was frequently present in the absence of anemia. However, the coexistence of both conditions does not necessarily establish a standardized biochemical diagnosis of iron-deficiency anemia, given that the original clinical classifications were not independently reassessed using predefined diagnostic criteria.

### 4.7. Implications for Practice, Research, and Policy

#### 4.7.1. Clinical Practice

For primary care teams, the findings support a structured reception pathway that combines epidemiologically informed screening with routine prevention, communication support, and planned review. The high frequency of vaccination need argues for making vaccine assessment a core element rather than a secondary task. The predominance of negative infectious composite results also supports restraint: testing should be connected to a clear risk indication and an actionable follow-up plan. The PAS model is consistent with a gateway function that transfers patients to standard primary care, rather than creating a permanent parallel service [6]. The hematological results further support incorporating basic anemia and iron-status review into this comprehensive assessment, particularly when age, sex, symptoms, dietary history, menstrual or obstetric history, or other clinical findings indicate increased risk.

#### 4.7.2. Research

The parent project’s mixed-methods design is a major opportunity to move beyond detection. The next research phase should evaluate whether infectious findings lead to completed treatment, whether vaccination indications lead to doses delivered and schedules completed, and whether the pathway improves continuity after the initial reception period. The qualitative component should examine barriers and facilitators to implementation and directly assess communication, understanding, navigation, acceptability, trust, and organizational health-literacy responsiveness. Longitudinal analyses will also be needed to distinguish need at entry from downstream outcomes. Future quantitative work should also distinguish laboratory-defined iron deficiency from broader iron-status abnormalities and cross-classify anemia with iron measures so that iron-deficiency anemia can be estimated directly.

#### 4.7.3. Policy

For policy makers, the results support investing in standardized but flexible primary-care entry processes for people seeking international protection. Standardization should concern the workflow—history, risk assessment, vaccination review, interpretation support, and follow-up—rather than imposing identical tests on every person. Such models can improve comparability and reduce unwarranted practice variation while preserving individualized risk assessment. This is aligned with calls for health systems that are inclusive, culturally responsive, and capable of collecting disaggregated data without turning origin into a deterministic risk label [2,5,6].

### 4.8. Strengths and Limitations

This study evaluates a real-world primary care reception pathway integrating medical and nursing assessments, preventive care, and coordinated follow-up. Its methodological strengths include the use of outcome-specific denominators, explicit handling of missing and non-requested tests, distinction between latent and active tuberculosis, and penalized regression to address sparse data. The analysis also distinguishes recorded clinical findings from standardized diagnostic estimates and avoids interpreting language or education as direct measures of individual health literacy.

Several limitations require equal emphasis. First, this is a preliminary analysis from one primary-care unit and one reception setting; the cohort is modest and some geographic strata are small, limiting precision and generalizability. Although participant records were incorporated consecutively according to the chronological order of their first PAS consultations, the interim analysis included only the 122 records entered into the research database by September 2026, while 128 additional records remained pending entry. Therefore, the findings may not fully represent the entire population attended by the programme. Furthermore, participant referral and access to the PAS were managed by CEAR through its routine appointment system, which may have influenced the composition of the analytical cohort. Household-level identifiers were not available in the analytical dataset, preventing assessment of potential within-household clustering and adjustment for correlations among participants from the same household. Second, the study is cross-sectional for the outcomes reported here and lacks an untreated, unstructured-care, or before–after comparator, so it cannot establish the causal ‘impact’ of the PAS. Third, infectious testing was intentionally risk-adapted. The composite therefore estimates detection within the programme, not population prevalence, and comparisons by geography may reflect both epidemiology and testing pathways. Fourth, the parent project remains in progress, and the present analytical cohort represents an interim data cut; later recruitment may change estimates.

Fifth, vaccination need was based on recorded clinical indications, not verified completion of catch-up schedules; the clinically important next outcome is vaccination delivered and completed. Sixth, the ≥4-consultation outcome is pragmatic and anchored to the modal three-visit pathway; it should not be interpreted as inappropriate use, and visit count is an incomplete measure of service intensity. Seventh, the grief analyses were restricted to adults with available questionnaire data, used dimension-specific complete cases, involved multiple exploratory comparisons, and relied on an operational 0–2 recoding because the original validation did not publish a unique numeric recoding scheme. Although interpreter assistance was available when needed, differences in language comprehension and interpretation may have affected the comparability of responses across participants. Exploratory internal consistency estimates in the present sample were limited, particularly for concern and loss of identity. These findings, together with the small domain-specific sample sizes and potential response-related selection bias arising from voluntary questionnaire participation, limit the interpretation and generalizability of the psychosocial results. Finally, individual health literacy, patient experience, acceptability, trust, and perceived communication quality were not directly measured. Those gaps are central objectives for the qualitative phase of the funded mixed-methods project. Anemia was analyzed using a pre-coded binary clinical variable, whereas iron status was recorded as a categorical clinical assessment. The classification of iron deficiency was based on serum ferritin concentrations, using the reference intervals provided by the clinical laboratory. However, the original clinical categories were retained and were not independently reassessed in the present analysis using predefined diagnostic criteria. The non-normal iron-status category combines clinically recorded iron deficiency and minor iron-related alterations; therefore, it should not be interpreted as the prevalence of iron deficiency. In particular, the category “minor iron-related alterations” does not represent a separately defined biochemical diagnosis.

Laboratory reference intervals for hemoglobin and iron-related parameters are provided in Supplementary Table S8, although these should not be interpreted as universal diagnostic cut-offs. Furthermore, C-reactive protein (CRP) was not systematically measured alongside ferritin, so a potential influence of inflammation on ferritin concentrations cannot be completely excluded. No pregnant women were included in the study population; therefore, pregnancy-specific reference thresholds were not applicable.

Although anemia and iron status were cross-classified (Supplementary Table S7), the recorded clinical categories do not, by themselves, establish a standardized biochemical diagnosis of iron-deficiency anemia. Finally, only 12 anemia events were observed, making the adjusted sex estimate necessarily imprecise, and the extreme estimate for Asia in the iron-status analysis reflects sparse-data separation rather than a stable regional effect.

The cross-classification of anemia and iron status (Supplementary Table S7) showed that 11 of the 12 participants with anemia (91.7%) were classified as having iron deficiency, whereas one had normal iron status. Conversely, 30 of the 41 participants classified as having iron deficiency (73.2%) did not present with anemia. These findings indicate that recorded iron deficiency was frequently present in the absence of anemia. Nevertheless, the co-occurrence of anemia and iron deficiency should not automatically be interpreted as a standardized biochemical diagnosis of iron-deficiency anemia, since the original clinical classifications were retained and were not independently reassessed in the present analysis. Finally, only 12 anemia events were observed, making the adjusted sex estimate necessarily imprecise, and the extreme Asia estimate for iron status reflects sparse-data separation rather than a stable regional effect.

## 5. Conclusions

In this preliminary cohort, vaccination indications and relevant infectious findings were documented through distinct clinical assessment processes, while hematological assessment identified additional clinical needs requiring individual etiological evaluation. These findings support interpreting the PAS as a structured pathway into and through primary care rather than as a programme focused primarily on imported-infection screening. The model combines risk-adapted screening, prevention, understandable communication, coordinated review, and transition to routine care. Differences across sociodemographic or geographic groups should be interpreted cautiously because the analysis is preliminary, cross-sectional, single-centre, and includes small strata. Individual health literacy was not measured, and the design does not permit causal attribution of outcomes to the PAS. The complete evaluation should determine whether identified needs translate into completed vaccination and treatment, continuity of care, and an experience of care that is understandable, accessible, and culturally responsive.

## Figures and Tables

**Table 1:** Sociodemographic and migration-related characteristics of the preliminary analytical cohort (N = 122).

Characteristic	Value
Age, years	26.0 (19.0–33.0); mean 27.1 ± 14.2
Age group	<18: 24 (19.7%); ≥18: 98 (80.3%)
Sex	Male: 87 (71.3%); Female: 35 (28.7%)
Geographical area	Sub-Saharan Africa 52 (42.6%); Latin America 42 (34.4%); Eastern Europe 15 (12.3%); Asia 10 (8.2%); Maghreb 3 (2.5%)
Background	Urban 87 (71.3%); Rural 35 (28.7%)
Family accompaniment	Travelled without family 72 (59.0%); With family 50 (41.0%)
Education, n = 103	No schooling 38 (36.9%); Primary 14 (13.6%); Secondary 37 (35.9%); University 14 (13.6%)
Main language	Spanish 42 (34.4%); Other language 80 (65.6%)
Migration route	Direct 48 (39.3%); Indirect 74 (60.7%)
Travel duration, months	1.0 (0.1–24.0); mean 16.5 ± 27.8
Time in Spain, months	11.0 (7.0–15.0); mean 11.1 ± 6.5
Time CEAR, months	1.5 (1.0–2.0); mean 1.5 ± 0.9
Duration of PAS process, days	28.0 (IQR: 14.0–42.0; range: 0–294)
PAS consultations	1: 5 (4.1%); 2: 16 (13.1%); 3: 66 (54.1%); 4: 22 (18.0%); 5: 11 (9.0%); 6: 2 (1.6%)

Values are n (%) unless otherwise stated. Continuous variables are shown as median (IQR), with mean ± SD also provided when informative. Percentages for education use the available-data denominator (n = 103), CEAR Torreblanca.

**Table 2:** Main quantitative outcomes, infectious components, vaccine-specific preventive needs, and secondary hematological findings.

Outcome/Component	n/N or n	Estimate/Interpretation
Panel A. Main outcomes		
Relevant infectious process detected	26/121	21.5% (15.1–29.6)
≥1 vaccination needs (HBV/MMR/varicella)	74/120	61.7% (52.7–69.9)
Extended PAS utilization (≥4 consultations)	35/122	28.7% (21.4–37.3)
Panel B. Components of the infectious composite		
Latent tuberculosis infection criterion	19	Detected cases; non-mutually exclusive
Active hepatitis B	4	Detected cases; non-mutually exclusive
Positive Schistosoma testing	3	Detected cases; non-mutually exclusive
HIV infection	1	Detected case
Positive stool parasitology	1	Detected case
Mycoplasma infection	1	Detected case
Panel C. Vaccine-specific needs		
Td-polio vaccination recommended *	112/120	93.3% (87.4–96.6)
Hepatitis B vaccination indicated	54/121	44.6% (36.1–53.5)
MMR vaccination indicated	37/120	30.8% (23.3–39.6)
Varicella vaccination indicated	29/120	24.2% (17.4–32.6)
Panel D. Secondary hematological findings		
Anemia	12/122	9.8% (5.7–16.4)
Non-normal iron status	56/120	46.7% (38.0–55.6)

HBV, hepatitis B virus; MMR, measles–mumps–rubella; PAS, Programa de Acogida Sanitaria. Infectious components are not mutually exclusive and were identified through risk-adapted testing; component counts should not be interpreted as prevalence estimates with a common denominator. For the vaccination composite, “No need” required all three vaccine indications to be evaluable and none to be indicated. For iron, “non-normal” combines the original categories “iron deficiency” and “minimal alteration”; “unsolicited” and “missing/non-evaluable” records are excluded from the denominator. Non-normal iron status should not be interpreted as a standardized biochemical definition of iron deficiency. * Td-polio vaccination was recommended when previous immunization status could not be reliably established because serological results and/or vaccination records were unavailable. This recommendation does not represent a serologically confirmed vaccination gap. The 95% confidence interval for Td-polio was calculated using the Wilson score method.

**Table 3:** Adjusted Firth logistic regression models for infectious detection and extended PAS utilization.

Adjusted Association	Events/N (%)	aOR (95% CI)	*p*-Value
Panel A. Relevant infectious process detected (N = 121)			
Age ≥ 18 years vs. <18 years		1.79 (0.30–19.09)	0.546
Female vs. male		2.17 (0.58–9.33)	0.249
Sub-Saharan Africa (Reference)	20/51 (39.2%)		
Latin America	4/42 (9.5%)	0.15 (0.03–0.55)	0.003
Maghreb	0/3 (0.0%)	0.17 (0.00–2.03)	0.182
Asia	0/10 (0.0%)	0.08 (0.00–0.96)	0.045
Eastern Europe	2/15 (13.3%)	0.25 (0.03–1.40)	0.120
Panel B. Extended PAS utilization ≥ 4 consultations (N = 122)			
Age ≥ 18 years vs. <18 years		0.42 (0.09–1.76)	0.235
Female vs. male		0.87 (0.28–2.66)	0.811
Sub-Saharan Africa (Reference)	20/52 (38.5%)		
Latin America	8/42 (19.0%)	0.29 (0.06–1.11)	0.072
Maghreb	1/3 (33.3%)	0.66 (0.05–5.64)	0.706
Asia	0/10 (0.0%)	0.02 (0.00–0.37)	0.004
Eastern Europe	6/15 (40.0%)	0.53 (0.09–2.71)	0.449
Travel with family vs. without family		0.94 (0.18–5.22)	0.942
Time in Spain, per 6 months		0.66 (0.38–1.06)	0.096

Panel A was adjusted simultaneously for age group, sex, and geographic area. Panel B was adjusted simultaneously for age group, sex, geographic area, family accompaniment, and time in Spain. Sub-Saharan Africa, age < 18 years, male sex, and travel without family are the reference categories where applicable. PAS, Programa de Acogida Sanitaria. The Asia estimates are based on a small subgroup (n = 10 overall) and should be interpreted as sparse-data signals rather than precise subgroup effects.

**Table 4:** Adjusted Firth logistic regression models for anemia and non-normal iron status.

Adjusted Association	Events/N (%)	aOR (95% CI)	*p*-Value
Panel A. Anemia (N = 122)			
Age, per 10-year increase		1.06 (0.67–1.58)	0.802
Female vs. male		4.75 (1.23–21.26)	0.023
Sub-Saharan Africa (Reference)	4/52 (7.7%)		
Latin America	4/42 (9.5%)	0.62 (0.11–3.08)	0.560
Maghreb	1/3 (33.3%)	4.05 (0.26–44.42)	0.289
Asia	2/10 (20.0%)	1.77 (0.21–11.27)	0.570
Eastern Europe	1/15 (6.7%)	0.43 (0.03–3.38)	0.440
Panel B. Non-normal iron status (N = 120)			
Age, per 10-year increase		0.46 (0.28–0.67)	<0.001
Female vs. male		1.41 (0.49–4.18)	0.527
Sub-Saharan Africa (Reference)	21/50 (42.0%)		
Latin America	19/42 (45.2%)	1.06 (0.38–2.87)	0.914
Maghreb	2/3 (66.7%)	3.18 (0.32–45.60)	0.327
Asia	10/10 (100.0%)	28.34 (2.67–3961.68)	0.003
Eastern Europe	4/15 (26.7%)	0.24 (0.05–1.03)	0.055

Models were adjusted simultaneously for age as a continuous variable per 10-year increase, sex, and geographical area; sub-Saharan Africa and male sex were the reference categories. The anemia model contained 12 events. The iron model excluded “unsolicited” and other non-evaluable records. The Asia estimate for non-normal iron status is based on 10/10 positive observations and is therefore extremely imprecise.

**Table 5:** Migratory-grief scores and exposure-specific adjusted associations.

Domain/Exposure	Descriptive or N	Adjusted Mean Difference (95% CI)	Interpretation/*p*
Panel A. Descriptive scores in adults			
Fear (0–12)	7.0 (3.0–9.0)	6.3 ± 3.3	Higher score = greater fear
Nostalgia (0–10)	7.0 (6.0–9.0)	6.9 ± 2.3	Higher score = greater nostalgia
Concern (0–8)	5.0 (4.0–6.0)	5.0 ± 1.6	Higher score = greater concern
Loss of identity (0–4)	0.0 (0.0–2.0)	0.7 ± 1.0	Interpret cautiously
Panel B. Exposure-specific adjusted models			
Family accompaniment: fear	N = 67	+1.29 (−1.70 to 4.29)	*p* = 0.392
Family accompaniment: nostalgia	N = 68	+0.34 (−0.97 to 1.65)	*p* = 0.606
Family accompaniment: concern	N = 68	−0.40 (−1.76 to 0.97)	*p* = 0.563
Family accompaniment: loss of identity	N = 67	−0.12 (−0.77 to 0.52)	*p* = 0.708
Secondary/university education: fear	N = 62	+1.82 (−0.10 to 3.74)	*p* = 0.063
Secondary/university education: nostalgia	N = 63	+1.17 (−0.15 to 2.49)	*p* = 0.081
Secondary/university education: concern	N = 63	−0.60 (−1.57 to 0.38)	*p* = 0.226
Secondary/university education: loss of identity	N = 62	−0.34 (−0.93 to 0.24)	*p* = 0.248
Latin America vs. sub-Saharan Africa: fear	N = 62	−0.63 (−3.28 to 2.02)	*p* = 0.635
Latin America vs. sub-Saharan Africa: nostalgia	N = 63	+0.57 (−0.69 to 1.82)	*p* = 0.370
Latin America vs. sub-Saharan Africa: concern	N = 63	−1.14 (−2.36 to 0.08)	*p* = 0.066
Latin America vs. sub-Saharan Africa: loss of identity	N = 62	−0.08 (−0.88 to 0.72)	*p* = 0.839

The adult grief subsample included 69 participants with at least one questionnaire response. Analytical sample sizes (n = 62–68) varied because each model required complete data for the corresponding grief-domain score, exposure, and adjustment covariates. Adjusted models included sex, age per 10 years, and time in Spain per 6 months. Exposure contrasts were travel with family versus without family, secondary/university versus none/primary education, and Latin America versus sub-Saharan Africa. Geographical comparisons were restricted to eligible participants from these two regions. The loss-of-identity factor should be interpreted cautiously because the original validation reported low internal consistency. No adjustment for multiple comparisons was applied.

## Data Availability

The datasets presented in this article are not publicly available because of ethical, legal, and privacy restrictions related to clinical-record data from a potentially identifiable and vulnerable population. Requests for data access should be directed to the corresponding author and will be considered in accordance with the ethics approval, applicable data-protection requirements, and data-custodianship conditions.

## Supplementary Materials

The following supporting information can be downloaded at: https://www.mdpi.com/article/10.3390/healthcare14213791/s1, Table S1. Univariable Firth associations with relevant infectious process detection. Table S2. Exploratory bivariate associations with any vaccination need (HBV/MMR/varicella). Table S3. Sensitivity analysis of number of PAS consultations using Poisson regression with HC3 robust standard errors. Table S4. Migratory-grief sensitivity models with additional adjustment for geographic area. Table S5. Exploratory bivariate distribution of anemia and non-normal iron status by sex, age group, and geographical area. Table S6. Infection-specific testing coverage and positive findings by geographical region. Table S7. Recorded iron status and cross-classification with anemia. Table S8. Laboratory reference intervals for hematological and biochemical parameters. Table S9. Completeness and classification of the composite vaccination-need outcome. Table S10. Time in Spain and at CEAR by PAS consultation count. Table S11. Duration of the Health Reception Programme (PAS), from the first recorded consultation to administrative closure of the programme. Table S12. Outcome-specific data availability in the interim analytical cohort. Table S13. Distribution of participants by recorded country or territory of origin (N = 122). Table S14. Sensitivity analysis of non-normal iron status using categorical age in multivariable Firth logistic regression. Table S15. Baseline characteristics of adult migratory-grief questionnaire respondents and non-respondents (N = 98). Table S16. Sensitivity analysis of the infectious composite restricted to latent tuberculosis infection, active hepatitis B, and HIV. Table S17. Exploratory internal consistency of migratory-grief questionnaire domains. Table S18. Sensitivity analysis of PAS utilization: alternative temporal measures in a common analytical sample (N = 121). Figure S1. CEAR service users, interim analytical cohort and outcome data availability.

## Abbreviations

Abbreviation	Definition
CEAR	Spanish Commission for Refugee Aid (Comisión Española de Ayuda al Refugiado)
CI	Confidence interval
ECDC	European Centre for Disease Prevention and Control
HBV	Hepatitis B virus
IQR	Interquartile range
LTBI	Latent tuberculosis infection
MMR	Measles–mumps–rubella
OR/aOR	Odds ratio/adjusted odds ratio
PAS	Programa de Acogida Sanitaria (Health Reception Programme)
SD	Standard deviation
STROBE	Strengthening the Reporting of Observational Studies in Epidemiology
WHO	World Health Organization
