# Supplementary material for: A Structured Health Reception Programme in Primary Care for Asylum Seekers: Preliminary Clinical, Preventive, Service-Use and Psychosocial Findings
Source: Healthcare (Basel). 2026 Nov 6;14(21):3791. doi: 10.3390/healthcare14213791 (PMC13762531; doi:10.3390/healthcare14213791)
Supplement: Supplementary file 1 [file healthcare-14-03791-s001.zip › healthcare-4626460-supplementary.pdf]

Supplementary Material

A Structured Health Reception Programme in Primary Care for Refugees and Asylum Seekers: Interim analysis of 122 participants

Table S1. Univariable Firth associations with relevant infectious process detection.

Characteristic	OR (95% CI)	p-value
Age ≥18 vs <18	5.51 (1.31–51.15)	0.016
Female vs male	0.71 (0.25–1.84)	0.496
Latin America vs sub-Saharan Africa	0.18 (0.05–0.51)	<0.001
Maghreb vs sub-Saharan Africa	0.22 (0.00–2.44)	0.247
Asia vs sub-Saharan Africa	0.07 (0.00–0.62)	0.011
Eastern Europe vs sub-Saharan Africa	0.28 (0.05–1.07)	0.065
Rural vs urban	4.27 (1.75–10.70)	0.002
Travel with family vs without	0.15 (0.04–0.45)	<0.001
Secondary/university vs none/primary education	0.28 (0.10–0.71)	0.007
Other language vs Spanish	3.35 (1.21–11.28)	0.018
Indirect vs direct migration route	1.01 (0.42–2.48)	0.987
Time in Spain, per 6 months	1.25 (0.86–1.85)	0.224

These analyses are exploratory and unadjusted. Strong dependence among geography, rurality, accompaniment, education, and language limits causal interpretation of individual estimates.

Table S2. Exploratory bivariate associations with any vaccination need (HBV/MMR/varicella).

Characteristic	Need, n/N (%) or median (IQR)	Global p-value
Age <18 years	19/24 (79.2%)	0.061
Age ≥18 years	55/96 (57.3%)	
Male	53/85 (62.4%)	0.838
Female	21/35 (60.0%)	
Sub-Saharan Africa	30/50 (60.0%)	0.243
Latin America	23/42 (54.8%)	
Maghreb	1/3 (33.3%)	
Asia	8/10 (80.0%)	
Eastern Europe	12/15 (80.0%)	
Urban	55/87 (63.2%)	0.675
Rural	19/33 (57.6%)	
Travelled without family	42/70 (60.0%)	0.706
Travelled with family	32/50 (64.0%)	
No/primary education	32/50 (64.0%)	0.314
Secondary/university education	27/51 (52.9%)	
Spanish main language	23/42 (54.8%)	0.325
Other main language	51/78 (65.4%)	
Time in Spain, months: no vaccine need	Median 12 (IQR 7–16)	0.140

Characteristic	Need, n/N (%) or median (IQR)	Global p-value
Time in Spain, months: ≥1 vaccine need	Median 10 (IQR 6–14)	

Categorical comparisons used Fisher exact tests; time in Spain used the Wilcoxon rank-sum test. No multiplicity adjustment was applied.

Table S3. Sensitivity analysis of number of PAS consultations using Poisson regression with HC3 robust standard errors.

Characteristic	Rate ratio (95% CI)	p-value
Age ≥18 vs <18	0.95 (0.76–1.19)	0.652
Female vs male	1.08 (0.90–1.29)	0.409
Latin America vs sub-Saharan Africa	0.88 (0.73–1.05)	0.151
Maghreb vs sub-Saharan Africa	1.05 (0.64–1.72)	0.850
Asia vs sub-Saharan Africa	0.84 (0.69–1.02)	0.086
Eastern Europe vs sub-Saharan Africa	1.00 (0.76–1.31)	0.998
Travel with family vs without	0.95 (0.78–1.16)	0.619
Time in Spain, per 6 months	0.97 (0.92–1.02)	0.215

This sensitivity analysis was used to assess robustness of the binary outcome of four or more visits; rate ratios, 95% confidence intervals, and p-values are reported.

Table S4. Migratory-grief sensitivity models with additional adjustment for geographic area.

Exposure/domain	N	Adjusted mean difference (95% CI)	p-value
Travel with family: fear	62	+1.26 (−2.72 to 5.25)	0.528
Travel with family: nostalgia	63	+0.60 (−1.17 to 2.36)	0.500
Travel with family: concern	63	−0.06 (−1.71 to 1.58)	0.940
Travel with family: loss of identity	62	−0.16 (−1.11 to 0.79)	0.735
Secondary/university education: fear	57	+2.68 (0.70 to 4.66)	0.009
Secondary/university education: nostalgia	58	+1.02 (−0.42 to 2.45)	0.162
Secondary/university education: concern	58	−0.58 (−2.10 to 0.94)	0.447
Secondary/university education: loss of identity	57	−0.34 (−0.99 to 0.30)	0.288

Models were restricted to sub-Saharan African and Latin American participants and adjusted for sex, age per 10 years, time in Spain per 6 months, and geographic area. Results are hypothesis-generating; no multiplicity adjustment was applied.

Table S5. Exploratory bivariate distribution of anemia and non-normal iron status by sex, age group, and geographical area.

Characteristic	n/N	% (95% CI)	Global p-value
**Panel A. Anemia**			
Sex: male	5/87	5.7% (2.5–12.8)	0.037
Sex: female	7/35	20.0% (10.0–35.9)	
Age <18 years	1/24	4.2% (0.7–20.2)	0.457
Age ≥18 years	11/98	11.2% (6.4–19.0)	
Geographical area: sub-Saharan Africa	4/52	7.7% (3.0–18.2)	0.329
Geographical area: Latin America	4/42	9.5% (3.8–22.1)	
Geographical area: Maghreb	1/3	33.3% (6.1–79.2)	
Geographical area: Asia	2/10	20.0% (5.7–51.0)	

Characteristic	n/N	% (95% CI)	Global p-value
Geographical area: Eastern Europe	1/15	6.7% (1.2–29.8)	
**Panel B. Non-normal iron status**			
Sex: male	39/85	45.9% (35.7–56.4)	0.842
Sex: female	17/35	48.6% (33.0–64.4)	
Age <18 years	18/24	75.0% (55.1–88.0)	0.003
Age ≥18 years	38/96	39.6% (30.4–49.6)	
Geographical area: sub-Saharan Africa	21/50	42.0% (29.4–55.8)	0.002
Geographical area: Latin America	19/42	45.2% (31.2–60.1)	
Geographical area: Maghreb	2/3	66.7% (20.8–93.9)	
Geographical area: Asia	10/10	100.0% (72.2–100.0)	
Geographical area: Eastern Europe	4/15	26.7% (10.9–52.0)	

Overall p-values are derived from Fisher's exact tests. For iron status, records marked as NOT REQUESTED and other non-evaluable records were excluded from the denominator. These analyses are exploratory and should not be interpreted in terms of causality.

Table S6. Infection-specific testing coverage and positive findings by geographical region

Region	Infection/test	Region N	Tested n	Positive n
Asia	Latent tuberculosis infection	10	10	0
Eastern Europe	Latent tuberculosis infection	15	12	2
Latin America	Latent tuberculosis infection	42	40	2
Maghreb	Latent tuberculosis infection	3	3	0
Sub-Saharan Africa	Latent tuberculosis infection	52	46	15
Asia	Mycoplasma	10	0	0
Eastern Europe	Mycoplasma	15	1	0
Latin America	Mycoplasma	42	2	1
Maghreb	Mycoplasma	3	1	0
Sub-Saharan Africa	Mycoplasma	52	3	0
Asia	Stool parasitology	10	4	0
Eastern Europe	Stool parasitology	15	2	0
Latin America	Stool parasitology	42	10	1
Maghreb	Stool parasitology	3	0	0
Sub-Saharan Africa	Stool parasitology	52	40	0
Asia	Schistosoma	10	0	0
Eastern Europe	Schistosoma	15	0	0
Latin America	Schistosoma	42	1	0
Maghreb	Schistosoma	3	0	0
Sub-Saharan Africa	Schistosoma	52	40	3
Asia	Active hepatitis B	10	10	0
Eastern Europe	Active hepatitis B	15	15	0
Latin America	Active hepatitis B	42	42	0
Maghreb	Active hepatitis B	3	3	0
Sub-Saharan Africa	Active hepatitis B	52	51	4
Asia	Active hepatitis C	10	10	0
Eastern Europe	Active hepatitis C	15	15	0
Latin America	Active hepatitis C	42	42	0
Maghreb	Active hepatitis C	3	3	0
Sub-Saharan Africa	Active hepatitis C	52	51	0
Asia	HIV	10	10	0
Eastern Europe	HIV	15	15	0
Latin America	HIV	42	42	0
Maghreb	HIV	3	3	0
Sub-Saharan Africa	HIV	52	51	1
Asia	Syphilis	10	10	0
Eastern Europe	Syphilis	15	15	0
Latin America	Syphilis	42	42	0

Maghreb	Syphilis	3	3	0
Sub-Saharan Africa	Syphilis	52	51	0

Screening was risk-adapted and not universal. Geographic regions were used for aggregation; individual testing decisions depended on country-specific epidemiology and clinical factors. Positivity among those tested should not be interpreted as population prevalence.

Table S7. Recorded iron status and cross-classification with anemia

Iron-status category	n	Evaluability
Normal	64	Evaluable
Minimal alteration	15	Evaluable; non-normal
Iron deficiency	41	Evaluable; non-normal
Not requested	1	Not evaluable
Missing	1	Not evaluable

Among the 120 participants with evaluable iron-status data, non-normal iron status was recorded in 56 (46.7%), including 41 (34.2%) classified as having iron deficiency. Iron-status categories were derived from the original clinical classifications recorded in the study database. The composite non-normal iron-status category included minor iron-related alterations and clinically recorded iron deficiency. These categories were based on the original clinical records and were not independently reassessed using predefined biochemical thresholds in the present analysis. Therefore, they should be interpreted as recorded clinical classifications rather than independently confirmed biochemical diagnoses.

Anemia	Normal	Minimal alteration	Iron deficiency	Not requested	Missing
No	63	15	30	1	1
Yes	1	0	11	0	0

Table S8. Laboratory reference intervals for haematological and biochemical parameters

Parameter	Women	Men	Girls	Boys
Haemoglobin (g/dL)	12–16	13–17.5	11.5–15.5	11.5–15.5
Ferritin (ng/mL)	30–204	30–275	15–150	30–275
Iron-binding capacity (µg/dL)	280–462	280–462	280–462	280–462
Transferrin (mg/dL)	200–400	215–365	250–380	200–400
Transferrin saturation (%)	20–50	15–45	15–45	20–50

Source: Reference intervals provided by the clinical laboratory for this study. These values represent laboratory reference intervals and should not be interpreted as universal diagnostic cut-offs.

Table S9. Completeness and classification of the composite vaccination-need outcome

Vaccination assessment	n (%)
All three indications evaluable	120 (98.4%)
Fewer than three indications evaluable	2 (1.6%)
Classified as having vaccination need	74/120 (61.7%)
Classified as having no vaccination need	46/120 (38.3%)

Percentages for evaluability are based on all 122 participants; percentages for vaccination-need classification are based on the 120 classifiable participants. All classifiable participants had all three indications evaluable. The original composite permitted a positive classification with at least one positive indication, even when other indications were unavailable.

Table S10. Time in Spain and at CEAR by PAS consultation count

PAS visits	Group n	Spain n	Spain median [IQR]	CEAR n	CEAR median [IQR]
1	5	5	16 [11; 16]	5	2 [2; 2]
2	16	16	13.5 [8; 18]	16	1.2 [1; 2]
3	66	66	10 [6; 14.8]	65	1.5 [1; 2]
≥4	35	35	10 [7; 13.5]	35	1 [0.8; 2]

Values are median [P25; P75]. Time in Spain and time registered at CEAR are different measures, not PAS follow-up duration.

Table S11. Duration of the Health Reception Programme (PAS), from the first recorded consultation to administrative closure of the programme.

Indicator	Result
Analytical cohort, n	122
Both dates available, n	120
Valid intervals included, n	120
Incomplete date information, n	2
Negative intervals excluded, n	0
Median duration, days (IQR)	28 (14–42)
Mean duration, days	39.17
Full range, days	0–294
Intervals of 0 days, n	5

Duration was calculated as the number of days between the first recorded PAS consultation and administrative closure of the programme, documented by issuance of the final health report. Administrative closure did not necessarily imply completion of all planned investigations. The summary is based on 120 participants with both dates available.

Table S12. Outcome-specific data availability in the interim analytical cohort

Outcome	Evaluable n	Non-evaluable n	Positive or category result
Relevant infectious process	121	1	26/121 (21.5%)
Any vaccination need	120	2	74/120 (61.7%)
PAS consultation count	122	0	≥4 visits: 35/122 (28.7%)
Anemia	122	0	12/122 (9.8%)
Iron status	120	2	Non-normal: 56/120 (46.7%)
Migratory grief (adult respondents)	69 of 98 answered ≥1 item	29 of 98 answered no items	Complete-case n varies by domain

Iron non-evaluability: one test not requested and one missing record. Infectious disease testing availability varied by condition (Table S6); the grief questionnaire response does not imply complete data for every dimension.

Table S13. Distribution of participants by recorded country or territory of origin (N = 122)

Country or territory of origin	n (%)	Analytical geographical region
Venezuela	31 (25.4)	Latin America
Mali	25 (20.5)	Sub-Saharan Africa
Senegal	18 (14.8)	Sub-Saharan Africa
Ukraine	15 (12.3)	Eastern Europe
Palestine	9 (7.4)	Asia
Colombia	7 (5.7)	Latin America
Gambia	3 (2.5)	Sub-Saharan Africa
Peru	2 (1.6)	Latin America
Sahara (as recorded)	1 (0.8)	Maghreb
Sahara (as recorded)	1 (0.8)	Sub-Saharan Africa
Algeria	1 (0.8)	Maghreb
Burkina Faso	1 (0.8)	Sub-Saharan Africa
Chile	1 (0.8)	Latin America
Ghana	1 (0.8)	Sub-Saharan Africa
Guinea (Conakry)	1 (0.8)	Sub-Saharan Africa
Honduras	1 (0.8)	Latin America
Morocco	1 (0.8)	Maghreb
Mauritania	1 (0.8)	Sub-Saharan Africa
Pakistan	1 (0.8)	Asia
Republic of the Congo	1 (0.8)	Sub-Saharan Africa
**Total**	**122 (100.0)**	**—**

Percentages are calculated using the full interim cohort (N = 122). Country and territory labels reflect the original clinical records; "Sahara" is retained as recorded and is not reassigned to a country. Totals may differ slightly from the sum of displayed percentages because of rounding.

Table S14. Sensitivity analysis of non-normal iron status using categorical age in multivariable Firth logistic regression

Characteristic	Adjusted OR (95% CI)	p-value
Age <18 vs ≥18 years	7.62 (2.10–35.01)	0.001
Female vs male	1.64 (0.59–4.80)	0.342
Latin America vs sub-Saharan Africa	0.56 (0.19–1.53)	0.264
Maghreb vs sub-Saharan Africa	2.01 (0.24–23.36)	0.513
Asia vs sub-Saharan Africa	12.44 (1.27–1671.30)	0.027
Eastern Europe vs sub-Saharan Africa	0.13 (0.02–0.68)	0.014

The sensitivity model included 120 participants (56 with non-normal iron status and 64 with normal iron status), and adjusted for sex and geographical region. Adults (≥18 years), males, and sub-Saharan Africa were the reference categories. Age was categorized as younger than 18 years versus 18 years or older, replacing the continuous age term in the primary model (Table 4). Penalized Firth logistic regression was used. The findings are exploratory and do not establish the shape of the age–outcome relationship. The small pediatric subgroup (n = 24), sparse age-by-region strata, and separation in the Asian regional category warrant cautious interpretation.

Table S15. Baseline characteristics of adult migratory-grief questionnaire respondents and non-respondents (N = 98)

Characteristic	Respondents (n=69)	Non-respondents (n=29)
Age, years, median (IQR)	27.0 (23.0–33.0)	33.0 (27.0–39.0)
Sex		
Male	53 (76.8%)	19 (65.5%)
Female	16 (23.2%)	10 (34.5%)
Geographical region		
Sub-Saharan Africa	41 (59.4%)	11 (37.9%)
Latin America	22 (31.9%)	9 (31.0%)
Maghreb	2 (2.9%)	1 (3.4%)
Asia	1 (1.4%)	3 (10.3%)
Eastern Europe	3 (4.3%)	5 (17.2%)
Area of origin		
Urban	42 (60.9%)	21 (72.4%)
Rural	27 (39.1%)	8 (27.6%)
Travel accompaniment		
Without family	54 (78.3%)	18 (62.1%)
With family	15 (21.7%)	11 (37.9%)
Educational level (available data: n=64 and n=26)		
No formal education	23 (35.9%)	6 (23.1%)
Primary education	9 (14.1%)	5 (19.2%)
Secondary education	23 (35.9%)	10 (38.5%)
University education	9 (14.1%)	5 (19.2%)
Missing, n	5	3
Main language		
Spanish	22 (31.9%)	9 (31.0%)
Other language	47 (68.1%)	20 (69.0%)
Migration route		
Direct	27 (39.1%)	12 (41.4%)
Indirect	42 (60.9%)	17 (58.6%)
Travel duration, months, median (IQR)	1.0 (0.1–24.0)	0.2 (0.1–24.0)
Time in Spain, months, median (IQR)	12.0 (8.0–16.0)	8.0 (5.0–10.0)
Time at CEAR, months, median (IQR)	1.0 (1.0–2.0)	1.3 (1.0–2.0)
Missing time at CEAR, n	0	1
Number of PAS consultations		
1	2 (2.9%)	1 (3.4%)
2	11 (15.9%)	3 (10.3%)
3	38 (55.1%)	15 (51.7%)
4	12 (17.4%)	6 (20.7%)
5	5 (7.2%)	3 (10.3%)
6	1 (1.4%)	1 (3.4%)

Values are n (%) unless otherwise indicated. Percentages for categorical variables are calculated within each response group using available observations. For educational level, denominators are 64 respondents and 26 non-respondents, excluding five and three missing records, respectively; missing counts are displayed separately and are not included in these percentages. Time at CEAR was available for 69 respondents and 28 non-respondents. Respondents were defined as adults who answered at least one migratory-grief questionnaire item; this does not imply complete data for each grief domain. Comparisons are descriptive, without formal hypothesis testing.

Table S16. Sensitivity analysis of the infectious composite restricted to latent tuberculosis infection, active hepatitis B, and HIV

Panel A. Testing coverage and restricted-composite findings by geographical region

Geographical region	Region N	LTBI tested	HBV tested	HIV tested	All three evaluable	Restricted events, n/N (%)
Sub-Saharan Africa	52	46	51	51	46	17/46 (37.0%)
Latin America	42	40	42	42	40	2/40 (5.0%)
Maghreb	3	3	3	3	3	0/3 (0.0%)
Asia	10	10	10	10	10	0/10 (0.0%)
Eastern Europe	15	12	15	15	12	2/12 (16.7%)
**Total**	**122**	**111**	**121**	**121**	**111**	**21/111 (18.9%)**

Panel B. Adjusted geographical associations in the same complete-case analytical sample (N = 111)

Geographical region	Original composite, adjusted OR (95% CI)	p-value	Restricted composite, adjusted OR (95% CI)	p-value
Sub-Saharan Africa	Reference	—	Reference	—
Latin America	0.15 (0.03–0.54)	0.003	0.0781 (0.0106–0.3599)	<0.001
Maghreb	0.16 (0.00–1.90)	0.164	0.1689 (0.0011–2.1299)	0.192
Asia	0.08 (0.00–0.87)	0.036	0.0655 (0.0004–0.8825)	0.038
Eastern Europe	0.29 (0.03–1.62)	0.166	0.2606 (0.0246–1.6305)	0.160

LTBI, latent tuberculosis infection; HBV, hepatitis B virus; HIV, human immunodeficiency virus; OR, odds ratio; CI, confidence interval.

Abbreviations: LTBI, latent tuberculosis infection; HBV, hepatitis B virus; HIV, human immunodeficiency virus; OR, odds ratio; CI, confidence interval.

The restricted composite was defined as the detection of at least one of the following conditions: LTBI, active hepatitis B, or HIV infection. Panel A includes participants for whom all three components were evaluable (111/122), among whom 21 (18.9%) had at least one positive finding. Under the less restrictive classification rule, 22 of 121 classifiable participants (18.2%) had a positive restricted composite, compared with 26 of 121 (21.5%) for the original six-component outcome.

Panel B presents Firth penalized logistic regression models adjusted for age group (<18 versus ≥18 years), sex, and geographical region. Both models were fitted to the same 111 participants to ensure comparability. Sub-Saharan Africa, age <18 years, and male sex were the reference categories.

This post hoc sensitivity analysis examined the robustness of geographical associations after excluding infectious conditions subject to more selective testing. Differences in diagnostic testing indications across geographical regions may persist. The findings are exploratory, and estimates derived from small regional strata or zero-event categories should be interpreted cautiously.

Table S17. Exploratory internal consistency of migratory-grief questionnaire domains

Domain	Items, n	Complete cases, n	Cronbach's α (95% bootstrap CI)
Fear	6	67	0.614 (0.448–0.719)
Nostalgia	5	68	0.672 (0.493–0.781)
Concern	4	68	0.472 (0.184–0.642)
Loss of identity	2	67	−0.006 (−0.436–0.381)

Cronbach's α was calculated using participants with complete item responses for each domain; 95% confidence intervals were obtained using 1,000 bootstrap resamples. The analysis is exploratory and does not constitute a psychometric validation of the questionnaire. The loss-of-identity domain comprises only two items, which showed negligible inter-item association in this cohort. The estimates reflect respondents with available data and do not address potential response-related selection bias.

Table S18. Sensitivity analysis of PAS utilization: alternative temporal measures in a common analytical sample (N = 121)

Characteristic	Time in Spain model, adjusted OR (95% CI)	Time at CEAR model, adjusted OR (95% CI)
Age ≥18 vs <18 years	0.41 (0.09-1.73)	0.41 (0.09-1.71)

Female vs male	0.92 (0.29-2.82)	1.04 (0.34-3.11)
Latin America vs sub-Saharan Africa	0.32 (0.06-1.22)	0.36 (0.07-1.39)
Maghreb vs sub-Saharan Africa	0.65 (0.05-5.55)	1.03 (0.09-8.64)
Asia vs sub-Saharan Africa	0.03 (0.00-0.38)	0.05 (0.00-0.61)
Eastern Europe vs sub-Saharan Africa	0.55 (0.09-2.78)	0.84 (0.15-3.93)
Travel with family vs without	0.85 (0.16-4.83)	0.84 (0.16-4.76)
Time in Spain, per 6 months	0.66 (0.38-1.06)	-
Time at CEAR, per month	-	1.12 (0.70-1.74)

Both models used Firth penalized logistic regression for four or more PAS consultations, with identical adjustment covariates other than the temporal measure, and were fitted to the same 121 complete cases. Time registered at CEAR represents duration at the reception facility, not PAS follow-up time. Estimates are exploratory, and geographical contrasts based on small strata should be interpreted cautiously. OR, odds ratio; CI, confidence interval.

Figure S1. CEAR service users, interim analytical cohort and outcome data availability

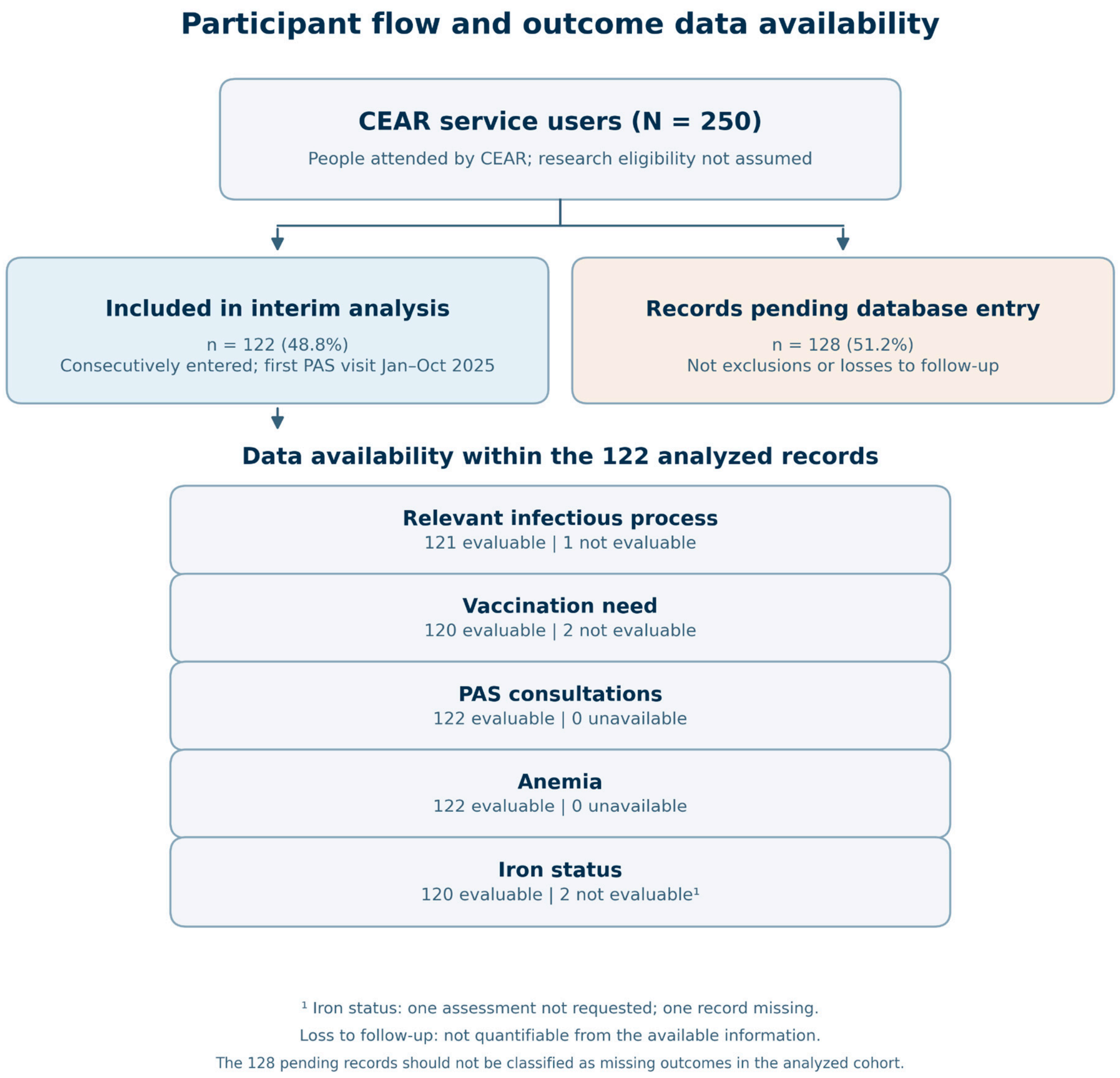

CEAR had attended 250 service users, of whom 122 records (48.8%) had been consecutively entered into the study database for the interim analysis and 128 records (51.2%) were pending database entry. The pending records are not exclusions or documented losses to follow-up, and are not counted as missing outcomes among the 122 analyzed participants. CEAR service use does not alone confirm study eligibility. Outcome-specific non-evaluable counts refer only to the 122 records in the interim cohort.
